# Evidence-Based Guide to Using Artificial Introns for Tissue-Specific Knockout in Mice

**DOI:** 10.3390/ijms241210258

**Published:** 2023-06-17

**Authors:** Elena McBeath, Keigi Fujiwara, Marie-Claude Hofmann

**Affiliations:** 1 Department of Endocrine Neoplasia & Hormonal Disorders, MD Anderson Cancer Center, Houston, TX 77030, USA; mhofmann@mdanderson.org; 2 National Coalition of Independent Scholars, Brattleboro, VT 05301, USA; keigifujiwara@gmail.com

**Keywords:** artificial intron, tissue-specific knockout, splicing, mRNA degradation, splicing prediction program

## Abstract

Up until recently, methods for generating floxed mice either conventionally or by CRISPR (Clustered Regularly Interspaced Short Palindromic Repeats)-Cas9 (CRISPR-associated protein 9) editing have been technically challenging, expensive and error-prone, or time-consuming. To circumvent these issues, several labs have started successfully using a small artificial intron to conditionally knockout (KO) a gene of interest in mice. However, many other labs are having difficulty getting the technique to work. The key problem appears to be either a failure in achieving correct splicing after the introduction of the artificial intron into the gene or, just as crucial, insufficient functional KO of the gene’s protein after Cre-induced removal of the intron’s branchpoint. Presented here is a guide on how to choose an appropriate exon and where to place the recombinase-regulated artificial intron (rAI) in that exon to prevent disrupting normal gene splicing while maximizing mRNA degradation after recombinase treatment. The reasoning behind each step in the guide is also discussed. Following these recommendations should increase the success rate of this easy, new, and alternative technique for producing tissue-specific KO mice.

## 1. Introduction

Knockouts and point mutations in mice are easy to perform with the CRISPR (Clustered Regularly Interspaced Short Palindromic Repeats)-Cas9 (CRISPR-associated protein 9) technique, but floxing a mouse this way has proven much more difficult. Using two guides and two donors to bracket an exon with loxP sites is quite inefficient, with an average success rate of less than 1% [1]. Several other methods for generating floxed mice by CRISPR-Cas9 have been developed to improve the low efficiency of correct dual-loxP insertion [2,3,4,5]. Microinjection into two-cell-stage mouse embryos or sequential injection/electroporation are more technically challenging, and I-GONAD, though a promising recent technique, requires new electroporation hardware. Using two guides with a long single-strand DNA (lssDNA) donor containing both loxP sites, with one at each end [6,7,8], appears to be 10 to 20 times more efficient than using two short donors [1]. The average efficiency of inserting the correct donor sequence for lssDNA methods ranges from 11 to 13%. However, particularly for floxing around long exons, lssDNA donors can be quite expensive to buy or time-consuming to make, and more importantly, the insert frequently contains unknown and unwanted mutations [9,10,11]. More recently, plasmid dsDNA donors were microinjected into both pronuclei during the zygote cell cycle phase, which has increased HDR. An average proportion of about 8% of correctly floxed mice were obtained from floxing 10 different chromosomal regions. Again, the authors noted that the method “is costly and time-consuming when preparing and maintaining an experimental environment in terms of both hardware and software” [12] despite using the much cheaper dsDNA plasmid template.

**Figure 1:**
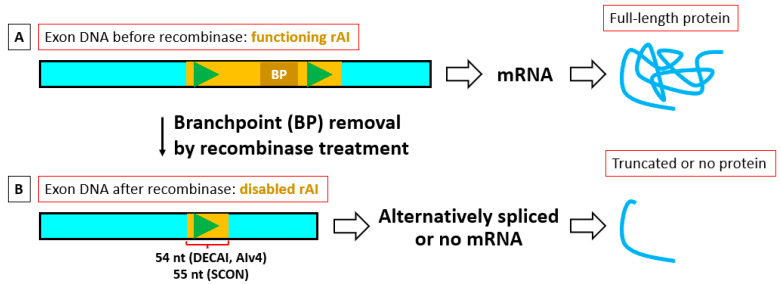
Overall strategy of how a recombinase-regulated artificial intron (rAI) is used to knockout functional protein production. (**A**) The recombinase-regulated artificial intron (rAI), when intact, completely splices out of the coding exon to obtain a normal mRNA and protein. (**B**) After removal of the rAI’s branchpoint by recombinase, the rAI will be unable to splice out, leading to alternative splice products. The alternatively spliced mRNA may be degraded, make truncated protein, or may do a combination of both. The recombinase-treated SCON rAI, at 55 nucleotides (nt), is more able to lead to multiple premature termination codons and thus better mRNA degradation than DECAI (DEgradation based on Cre-regulated Artificial Intron) or Alv4 at 54 nt (divisible by 3). Exon DNA is in light blue, rAI DNA in yellow, branchpoint (BP) dark yellow, loxP green triangles, and protein in blue.

**Figure 2:**
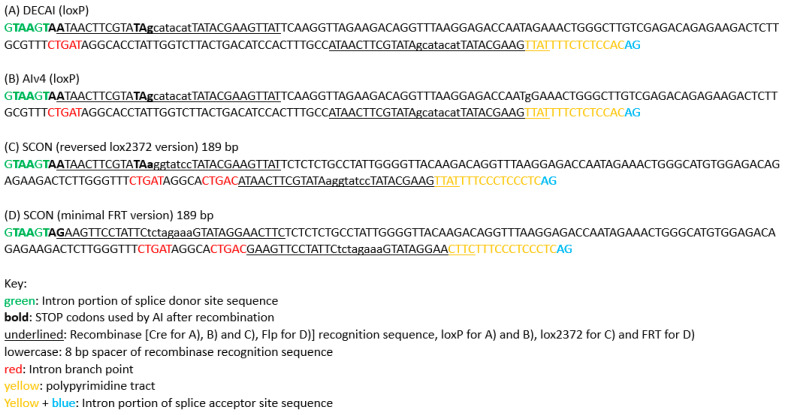
Recombination-regulated Artificial Intron (rAI) sequences. (**A**) The sequence of the DECAI artificial intron designed by Burkstummer’s group (Guzzardo et al. [13]). (**B**) The sequence of the Alv4 artificial intron used by Pelletier’s lab (Cassidy et al. [15]). The sequence differs from DECAI by a change of A to G in the 75th nt (75A>G). (**C**) The sequence of the SCON (Short Conditional intrON) artificial intron version with loxP, designed by Koo’s lab (Wu et al. [14]). (**D**) The sequence of the SCON artificial intron version with FRT, designed by Koo’s lab (Wu et al. [14]). The FRT version of SCON has only two STOP codons in different frames. If the third frame is needed, SCON must depend on a downstream endogenous premature STOP codon for truncating the protein and for nonsense-mediated decay (NMD) to occur once its branchpoints are removed by the Flp recombinase.

A few years ago, a different approach to floxing a gene was taken by Guzzardo et al. [13]. Using human stem cell lines, they inserted a small artificial intron via CRIPSR into an exon found in all isoforms of the protein intended for KO. They called this method DECAI (DEgradation based on Cre-regulated Artificial Intron). The 201-base-pair (bp) DECAI recombinase-regulated artificial intron (rAI) has loxP sites on each side of its branchpoint and, without Cre, splices itself out of the mRNA, leading to normal protein production (Figure 1 and Figure 2A). The intron also has three stop codons in different frames, so when the branchpoint sequence is removed by Cre, one of the stop codons is put in-frame. With proper placement of DECAI in the gene, this can lead to knockout of protein function through protein truncation, degradation, and/or degradation of its mRNA, although perhaps not usually through the mechanism postulated by Gezzardo et al. [13].

Due to needing only one guide and a small donor, the rAI method is easier, faster, and less expensive than the tissue-specific KO techniques that have been published for mice so far. For these reasons, two other groups, namely Wu et al. [14] and Cassidy et al. [15], have taken this approach, successfully applying the technique to mice. Cassidy et al. used an rAI almost identical to DECAI, i.e., AIv4 (Figure 2B), to flox a single gene. Wu et al. developed their own 189 bp rAI called SCON (Short Conditional intrON) (Figure 2C,D) and inserted the optimized version into 13 different genes. Although its sequence is different, SCON has the same features (stop codons in different frames and branchpoint(s) bracketed by two loxPs) as the DECAI intron, but unlike DECAI, SCON’s insertion appears to cause little to no reduction in gene product, at least in the single-exon-enhanced green fluorescent protein (eGFP) gene it was tested in. Both groups had a 100% success rate in placing their rAI into a target gene, and each showed one gene correctly spliced out. They both suggested a set of rules to follow when planning where to insert the artificial intron. They also pointed out some limitations to the method. 

Years ago, we also attempted to make a floxed mouse using the DECAI intron approach. We followed the splicing rules that we could find at that time as well as using an online prediction program to confirm good splicing. However, our project failed; the rAI did not splice out correctly, instead causing a global knockout. This is because we had broken one of the rules, namely that one should try inserting the rAI at least 100 bp away from the upstream, 5′ end of the exon (Query 2). When we performed that experiment, we had not yet come across any evidence in the literature for this rule. The splicing prediction program we used had not picked up on the error because it was based on an older, much less reliable machine learning algorithm than those available today.

Despite the high success rate of the two published groups, we noted that some other labs are also having difficulty with the rAI method. We offer here a detailed guide meant to increase the odds of successful splicing and recombinase-induced knockout (KO). It is a list of rules and suggestions compiled from a recent literary search on splicing and mRNA degradation as well as from combining some of the rules from the two above-mentioned papers for successful floxing by the rAI method. Use of the newer splicing prediction programs such as Spliceator, which are based on deep learning, are integral to the approach. Because there are many factors involved in splicing and because how these factors interact is very complicated or still unknown, the deep-learning- and neural-network-based programs that are capable of making non-linear, complex correlations are more accurate in their splice predictions than other older machine learning programs. We expect many future advances in splicing prediction but hope that in the interim, this guide will improve the chances of successful gene floxing with rAIs and tissue-specific loss of function after recombinase treatment. 

## 2. Data Analysis

**Figure 3:**
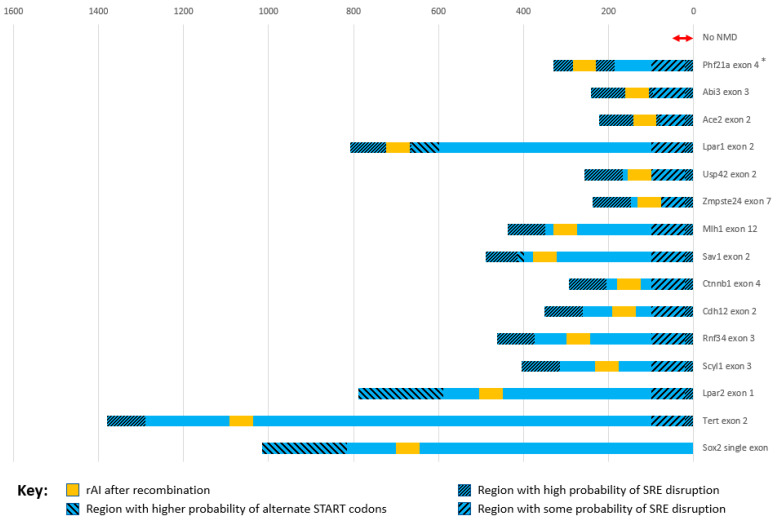
rAI placement within the exon, from published rAI containing mouse genes. The first ~100 nt in the exon from either end may have splicing regulatory elements (SREs) whose disruption will affect splicing, particularly those within the first 10–20 nt from either end. However, the ~80 nt sequence (wide stripes on right end) beyond the 20 nt at the 3′ splice donor site end has a lower probability of affecting splicing than at the 5′ end (narrow stripes). Because the rAI can act to significantly increase the distance of SREs from the exon end, the further it is placed into the striped region, the greater the probability of splicing dysfunction. In addition, on the 3′ end, the last 50–55 nt (double-headed red arrow) are critical for NMD, and it is recommended to avoid this region for rAI insertion. For exons at or close to the normal start site, rAI placement beyond the first 200 nt (wide stripes on left end) after the start codon is recommended to avoid transcription re-initiation at alternative start sites. Exons are in blue, rAI in yellow, and regions where rAI placement should be avoided have diagonal stripes. Horizontal units in nts; * from our unpublished data.

To devise some of the guidelines for the best placement of the rAI in the gene, we used published sequence data and our own data. Data reported in the “gRNAs and repair templates used in SCON mouse generation” section of the Supplementary Materials in Wu et al. [14] were used to reconstruct the rAI-inserted gene sequence for each gene. The gene sequence expected after recombinase treatment was also constructed. The same was performed using the donor insert information in the Methods section from Cassidy et al. [15]. We included our own construct, which has DECAI inserted in the MAG-R sequence, between the 46th and 47th base pair in exon 4 of the mouse Phf21a gene. The wild-type sequence, the rAI-inserted sequence, and the recombinase-shortened sequence of each gene were all analyzed with the Spliceator prediction program [16] (http://www.lbgi.fr/spliceator/, last accessed 8 March 2023), and the reliability score was determined for the rAI splice sites, the splice sites of the exon containing the rAI, the upstream exon’s donor splice site, and the downstream exon’s acceptor splice site. The splice site scores predicted by Spliceator for the exon in which the rAI resides are listed in Table 1. Figure 3 gives a visual representation of each exon containing a recombinase-treated rAI, showing exon length, rAI placement, and the regions where rAI placement should be avoided.

## 3. Guideline

There are two necessary conditions to make floxing by rAIs work. First, the rAI must splice out of the gene with no or minimal perturbation of the gene and protein function. Second and just as important is that there must be essentially no functional protein made after recombinase treatment. Listed below are instructions we propose for floxing a mouse gene with rAIs to achieve these goals. After describing in question from each issue that must be addressed, we provide background, justifications, and discussions that support the proposed steps to take in response.

## 4. Query 1: Into Which Exon Should One Insert the rAI?

**Figure 4:**
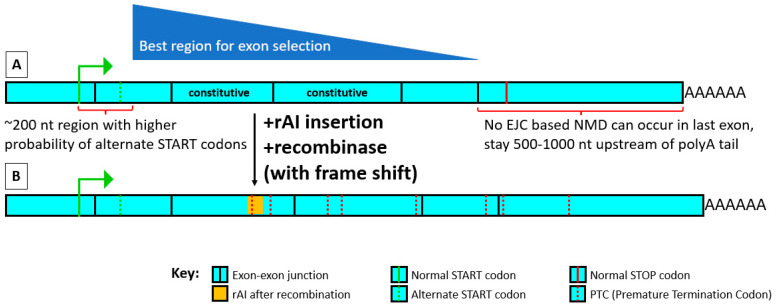
Spliced mRNA before and after rAI insertion and treatment with recombinase. Idealized mRNA after splicing, (**A**) before any insertion, and (**B**) after rAI insertion, followed by recombinase treatment so that the rAI no longer has its branchpoint and is unlikely to splice itself out. It is best to choose a constitutive exon in which one can follow the rules for rAI placement within an exon and one that is close to but not within 200 nt downstream of the start codon and is upstream from another constitutive exon. This preferred location is shown with the dark blue triangle. The closer to but not within 200 bp of the start codon, the greater the chance of mRNA-degradative processes occurring after recombinase treatment. In this example, an rAI non-divisible by 3, such as SCON, is demonstrated, causing a frameshift leading to multiple downstream premature termination codons (PTCs). This allows for a greater chance of mRNA degradation through NMD and by creating a long 3′ untranslated region (long 3′ UTR).

When selecting an exon, the main goal is to choose one in which the rules for placement within an exon can be followed while its placement within the mRNA maximizes the extent of degradation after recombinase treatment through processes such as nonsense-mediated decay (NMD) and long 3′ untranslated region (long 3′ UTR) generation (Figure 4).

*Start with constitutive exons whose alteration or loss is known to KO protein function. If not known, try using an exon producing a nonfunctional truncated frame-shifted protein that is as upstream as possible, at least 500–1000 nucleotides (nt) upstream from the mRNA’s polyA tail, to maximize various mRNA-degradative processes working after recombinase treatment. (Figure 4A)*

An exon with an essential function or that has previously been shown to cause KO of a protein when mutated is likely a good candidate. If no useful information specific to a protein is available to help select an appropriate exon, one can place the rAI in a more upstream exon. Any truncated protein made after rAI recombinase treatment that is not degraded will then be shorter and hopefully less likely to have a functional effect. The most 5′ exons [15] or an exon somewhere in the first third [13] or half [14] of the gene coding sequence have been recommended as rAI insertion sites.

Despite having no normal function, even a highly truncated protein may still affect cell physiology [17]. Therefore, one may also try rAI placement that increases the probability of mRNA-degradative processes occurring. In the presence of a premature termination codon (PTC), two major mechanisms of mRNA decay are known to occur. One mechanism depends on a splice junction forming an exon junction complex (EJC) mark downstream from the PTC and the other on the length of untranslated region following the PTC. The first is called nonsense-mediated decay (NMD), while the second refers to the increase in mRNA degradation seen with long 3′ UTRs. 

The further upstream the rAI-containing exon is, the gradually less effective NMD is likely to become (PTC to normal stop codon rule [18]), and the further away a normal stop codon is from the polyA tail, the more likely it is that readthrough can occur [19], which may also apply to the rAI’s PTCs. However, if readthrough does not occur, a longer 3′ UTR will be generated, which increases the probability of degradation by non-NMD processes [19]. One can take advantage of both NMD and long 3′ UTR-degradation processes by inserting the rAI further upstream and positioning the rAI in the exon to cause a frameshift after recombinase treatment. This usually generates numerous downstream PTCs and increases the length of the 3′ UTR, which can greatly increase both the chance of NMD and degradation by the presence of a long 3′ UTR. This has the added advantage that if degradation of mRNA and protein does not occur or occurs incompletely, the truncated product will be shorter and may therefore have less of an effect on the cell.

Various sequences in the 3′ UTR can both stabilize or destabilize it (e.g., ARE sequences [20,21]), so it is difficult to suggest a precise length to select to ensure adequate degradation. However, Hurt et al. [22] found that mouse 3′ UTRs of over 800 nt in length were more likely to be degraded by the long 3′ UTR mechanisms than by NMD versus short (50–350 nt) 3′ UTRs. Thus, we suggest searching upstream, if possible, by at least 500–1000 nt or more in the spliced RNA from the end of the gene when selecting an appropriate exon for rAI insertion.

*Start by using an exon over 150 bp in length, and over 200 bp is better, but do not insert the rAI within 200 bp of the start codon (the start-proximal rule) or in the last exon.*

An exon larger than 150–200 bp is suggested in order to more easily be able to follow the rules described in Query 2 below detailing where in the exon the rAI should be placed.

Once the branchpoint is removed from the rAI, the PTCs created by its internal stop codons and by frameshift should be downstream at least 200 nt or more, if possible, from any potential start codon. This is in order to prevent ribosomal rebinding [23] and to allow NMD to occur (start-proximal NMD evasion rule [24]).

Given how NMD works in mammals [25], the last exon should usually not be used. This is because an exon junction complex (EJC) mark is formed at each newly spliced exon-exon junction and is usually needed for NMD to occur. However, there is no exon–exon junction downstream of the last exon; therefore, no EJC mark can form, and there will be nothing to trigger NMD (last exon rule of NMD evasion [24]).

Intron-less proteins, with only one exon, have no splice sites, so NMD cannot occur by EJC marking. In these genes, the position of the rAI after removal of its branchpoint should still be frame-shifted to produce multiple PTCs. At the same time, it should be placed as upstream as feasible to increase the chance of long 3′ UTR-induced degradation and/or to make a non-functional truncated protein.

*We suggest choosing an exon in which the rAI can be positioned to create the greatest number of PTCs within 400 nt upstream of a splice junction after recombinase treatment. (Figure 4B)*

We define splice sites as “strong” if they score high in Spliceator, corresponding to the reliability scores of constitutive exon splice sites, while those that have a “weak” score are lower in Spliceator, at the level associated with alternative splice sites. Both the splice donor and acceptor sites of the rAI, which are quite strong, can function after removal of the branchpoint, although no longer efficiently with each other [15]. If the rAI is placed close to the exon’s 3′ end so that there are no strong cryptic splice sites between the rAI’s splice donor and the exon 3′ end, the rAI splice donor site (SDS) may very likely splice primarily with the downstream exon’s splice acceptor (see Query 2 discussion). If the rAI is positioned so that splicing with the downstream exon causes a frameshift producing multiple PTCs within 400 nt of an EJC, efficient degradation of the mRNA by NMD will likely follow. 

*If the immediate upstream and downstream exons are also constitutive, this may help reduce the number of alternatively spliced products after rAI branchpoint removal.*

When the immediate upstream and/or downstream exons are not constitutive, an increased variety of splice products are likely to be made, leading to a higher chance of generating full-length protein with indels. It may become harder or impossible to position the rAI so that all the feasible splice products made after branchpoint removal become frame-shifted.

## 5. Query 2: Where in the Exon Should the rAI Be Inserted?

**Figure 5:**
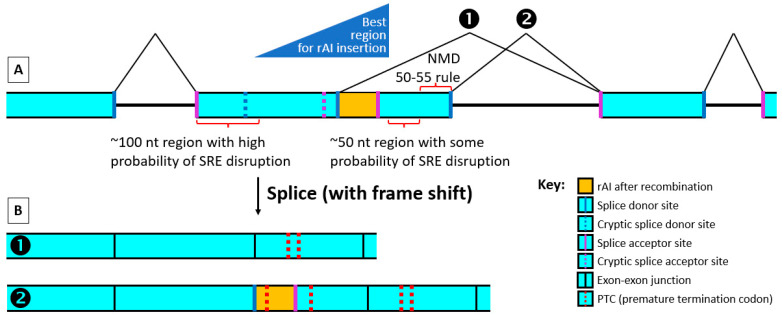
Splicing of mRNA with recombinase-treated rAI. Idealized mRNA after rAI insertion and recombinase treatment (**A**) with exons and introns before splicing and (**B**) with exons only after splicing. The dark blue triangle shows the region best for rAI insertion within a constitutive exon that will most likely lead to proper splicing before recombinase treatment. It is best to stay away from the first ~100 nt and the last ~100 nt in an exon. We also suggest moving to the right as far as feasible within this region in order to avoid having cryptic splice acceptor sites within the exon downstream of the rAI donor site. Because the rAI splice donor is very strong, it may splice with the downstream exon’s splice acceptor site (1). If the endogenous exon’s splice donor site is stronger, it may splice as usual with the downstream exon’s splice acceptor (2), or a combination of both may happen. By using an rAI non-divisible by 3, such as SCON, and with proper placement, a frameshift can occur in both cases, increasing the chance of multiple PTCs and mRNA degradation. SRE, splicing regulatory element; NMD, nonsense-mediated decay.

The objective in this case is twofold. The first is to position the rAI within the exon to avoid disrupting normal splicing before recombinase treatment. At the same time, the rAI should be positioned so that after recombinase treatment, a frameshift occurs (Figure 5).

*Whenever possible, insert the rAI at least 100 bp away from the upstream 5′ end of the exon. If it is less than 100 bp away from that end, the further away, the better. It may also be possible to use an up-to-date splice prediction program such as Spliceator to find locations within the 100 bp 5′ end where the predicted reliability of the endogenous splice sites in the vicinity of the rAI does not significantly change both before and after rAI branchpoint removal.*

Splicing regulatory elements (SREs) within the exon may be disturbed by the insertion of the rAI, causing unintended changes in how splicing occurs in the vicinity of the rAI. This includes splicing not only of the rAI itself but also the exon containing the rAI and nearby exons. Because an rAI is relatively large (~200 nt), insertion near one end of an exon will significantly displace SREs on the far side of that end, moving these sequences around 200 nt further away from the exon end. The closer the rAI gets to the exon end, the more likely it is that disruption of normal splicing will occur. For example, although the exonic SREs on the first ~50 nt beyond the splice sites have little effect on alternative splicing for that exon, they have a major effect on whether a neighboring exon will be included or skipped [26]. Splicing catalysis does not occur until PolII has transcribed 26–129 nt downstream into the exon from the splice acceptor site (SAS) [27]. The rate at which PolII moves through this region in turn controls the ratio of splicing at that acceptor versus one further downstream. This suggests that to be safe, one should insert the rAI at least 130 bp downstream of the acceptor. However, after testing from 20 to 600 nt sized search windows around the splice site, Scalziti et al. [16] found that the sequence beyond about 100 nt from the splice site provide very little further predictive value (Figure 6 in [16]). 

When we inadvertently broke the 100 bp upstream rule by placing DECAI 46 bp from the upstream 5′ end of constitutive exon 4 in the mouse Phf21a gene, it caused the DECAI splice acceptor to splice not with its splice donor but instead incorrectly splice with either the upstream exon splice donor or several cryptic splice donors in the upstream intron. The 5′ acceptor of the exon containing the rAI was no longer used, and its Spliceator score, which can go from 0 to 1, dropped almost 5%, from 0.981 to 0.935 (Table 1, Exon SAS; see also Query 4).

*Stay at least 50–55 nt, preferably 100 nt, from the downstream 3′ end of the exon while remaining close to this end.*

Although the chance of disrupting splicing beyond 50 nt is significantly lower on the 3′ end of the exon than the 5′ end [16], it is safer to position the rAI at least 100 nt upstream of the 3′ end whenever possible. As done for the SDS, one can check with an up-to-date splice prediction program such as Spliceator for minimal changes in the SAS splicing strength.

After a recombinase such as Cre cuts out the rAI’s branchpoint, the stop codon in the rAI that becomes in-frame or any other PTC created elsewhere by the shortened rAI causing a frameshift must be at least 50 nt upstream of an exon–exon junction for most NMD to occur (50–55 nt rule [28]). An exon junction complex (EJC) mark is ordinarily formed within this 50–55 nt region in the first 20–24 nt upstream of the junction by splicing at each exon–exon junction. The ribosome from a pioneering round of translation will knock off these EJC marks unless a PTC is present upstream of one of the EJCs. In that case, the EJC remains and usually will, along with the ribosome at the premature stop codon and several other proteins between, activate the NMD machinery. This is the primary way NMD occurs in metazoa, but other less-well-understood factors also influence NMD [25,29].

In long exons (>400 nt), PTCs become less and less efficient in inducing NMD the further upstream they are from the 50–55 nt position (long exon rule [24]). When depending on NMD for KO, we recommend that for long exons, the rAI be positioned to keep the most likely first PTCs produced by rAI branchpoint removal at least 50–55 nt upstream but still within 400 nt of an exon’s downstream 3′ end.

*While following the other rules, we suggest inserting the rAI as close as possible to the downstream 3′ end of the chosen exon such that splicing between the rAI donor site and the downstream exon SAS causes a frameshift. Try to choose the frame that causes the most PTCs within 55–400 nt of a downstream exon’s splice junction to increase the chance of efficient mRNA degradation.*

Although greatly reduced in their ability to splice with each other, the sequence of the rAI’s donor site and acceptor site with its polypyrimidine tract are unaffected by removal of the rAI branchpoint, and as both seen and stressed by Cassidy et al. [15], it is still possible for each to splice elsewhere. 

The rAI SAS can do so either by using cryptic upstream branchpoints, if present, or by branchpoints created at the rAI donor end upon its insertion into the gene. Most branchpoints occur within the first 10–50 nt upstream of the intron splice acceptor, while a few can be found several 100s of nts upstream or very close to the acceptor [30,31]. Thus, although the mammalian branchpoint sequence is highly degenerate [32], and there is always a possibility that the rAI splice acceptor will use a cryptic upstream SDS within the exon, the probability should be low. The use of a program such as Spliceator can point out possible upstream cryptic SDSs within the exon that might be of concern.

The rAI SDS can splice using downstream cryptic branchpoints and acceptors within the exon or the branchpoint and acceptor of the downstream exon. Comparing Spliceater splice site predictions with the actual splice sites used in a gene shows that the normal SDS downstream from the normal SAS is also almost invariably the highest-scoring donor site between the two. Predominantly, it is also the highest-scoring donor site to the next downstream acceptor site. Actual acceptor sites, on the other hand, more frequently are not the highest-scoring between either the normal upstream or downstream donor sites.

Not enough is known at this time to predict exactly how splicing will be altered in mice by the recombinase-treated rAI. However, we know that the rAI SDS sequence’s Spliceator reliability scores are very high, usually higher than that of the exon’s normal SDS, and splicing by an acceptor far from a branchpoint is rare [30,31]. Therefore, if the rAI is placed beyond any possible downstream cryptic acceptor in the exon, it seems likely that a major, and perhaps the major, splice product will have a fusion between the rAI SDS and the SAS of the downstream exon (Figure 5B(1)). Supporting this view, mutations in branchpoints that destroy their function typically result in downstream exon skipping [30]. The equivalent of the downstream exon in this case would be the second “mini-exon” produced when the rAI splits the exon into two “mini-exons”. 

Occasionally, two splicing donor site scores are very close in value. In such cases, the lower-scoring site can be chosen as the actual splicing donor site. Thus, the rAI-containing exon SDS may sometimes be chosen, despite having a lower score, to splice with the downstream exon, leaving the recombinase-shortened rAI as an insert (Figure 5B(2)).

Moving the rAI closer to the downstream end of the exon should also help reduce the chance of the rAI’s SAS using the upstream endogenous intron’s branchpoint.

Cassidy et al. [15] made a crucial point: the rAI “should be inserted such that potential splicing events driven by the” rAI “splice donor with downstream exon(s) results in an out of frame transcript”. As discussed above, there are two most likely outcomes of an rAI positioned close to the exon’s SDS downstream end: Either (1) the rAI donor splices with the downstream exon’s acceptor, or (2) the exon’s donor splices as usual with the downstream exon’s acceptor, generating an mRNA containing the shortened rAI with its 3 stop codons. We suggest positioning the SCON rAI SDS such that it will produce a frameshift if the rAI donor splices with the downstream exon SAS. This way, since the recombined SCON is not a multiple of 3 in length, whether the rAI’s SDS or the SDS of the exon is used to splice with the downstream exon (Figure 5), a frameshift will occur. With rAI positioning to ensure that this splicing event produces a frame-shifted product, multiple PTCs should be produced, greatly increasing the probability of mRNA destruction by NMD and the presence of a long 3′ UTR. There are two out-of-frame positions. If possible, choose the one that gives the greatest number of PTCs within 55–400 nt of a downstream exon’s splice junction.

*Given that the sequences at the ends of the DECAI, AIv4, and SCON inserts conform to the major class U2 introns, start by placing the rAI between the G and R of a MAG-R sequence (equivalent to (C/A)AG-(G/A)) that can lead to highly efficient splice donor and acceptor site sequences.*

It is key that splice SDS and SAS sequences produce a strong splice site to ensure robust, uniform splicing out of the rAI. This can be achieved by matching a consensus sequence. DECAI, AIv4, and SCON all have at their ends the correct intron donor and acceptor site sequences found in the canonical mammalian SDS with the consensus sequence of MAG|GTRAGT (equivalent to (C/A)AG|GT(A/G)AGT) and acceptor site consensus sequence of CAG|G, which are found in about 99% of splice sites [33]. The three bases at the SDS end of an exon modulate the strength of the splice site [34], so using a non-consensus sequence for the rAI can lead to a weaker SDS and a greater number of alternatively spliced products both before and after branchpoint removal. Thus, although other configurations can exist, one should first try to insert these rAIs between the G and R of a MAG-R sequence in the chosen exon to generate the canonical donor and acceptor sequences.

However, a strong splice site can be formed by other, non-canonical sequences. This is because (1) for all three rAIs, the rAI intron donor sequence complements the initial binding small nuclear RNA (snRNA) U1 sequence perfectly out to the 7th position, allowing the three exonic nucleotides to be a little less complementary [35], and (2) other factors such as exonic and intronic splicing enhancers and silencers (ESSs, ESEs, ISSs, and ISEs), heterogeneous ribonucleoproteins (hnRNPs), and serine- and arginine-rich proteins (SRs) all affect the rAI’s splicing donor strength. At present, Spliceator can be used to check if insertion into a non MAG-R site will likely create strong rAI splice donor and acceptor sites.

## 6. Query 3: Which rAI Should One Use?

*Between DECAI, AIv4, and SCON, SCON will usually make it easier to produce out-of-frame splice products after branchpoint removal, which leads to better NMD and is the first choice. SCON has the added advantage that it has been shown that loxP can be switched out for the Flp recombinase recognition sequence, FRT.*

Both Wu et al. [14] and Guzzardo et al. [13] found that DECAI insertion frequently reduced the endogenous level of gene expression, while Wu et al. saw that SCON insertion appeared to have little to no effect on eGFP gene expression in various cell lines and vertebrate species. However, alterations on gene expression levels by SCON in other genes were not directly tested.

After recombinase treatment, the Cre-shortened form of DECAI and AIv4 [15] is 54 nt, which is divisible by 3. This means that only one readthrough of the rAI’s in-frame stop codon is required to allow the mRNA of that shortened rAI to survive and produce a truncated product containing the shortened rAI insert, with no further chance of standard NMD occurring. Even if there is a long 3′ UTR, it may not have sufficient time to start the degradation process before the next random readthrough. On the other hand, at 55 nt, recombinase-shortened SCON is not divisible by 3 and thus will be downstream frame-shifted and by chance is likely to produce multiple downstream PTCs at which both a truncated form of the protein will be made, which could lead to NMD (Figure 1B). 

These splicing pathways might be of little importance, as the major spliced forms may come from the rAI SDS splicing with SASs further downstream, particularly with the downstream exon SAS, as seen by Cassidy et al. [15]. In that case, regardless of which rAI is used, it should be placed such that after splicing with the downstream exon acceptor, the mRNA is out of frame.

Wu et al. [14] showed that FRT could be used in place of loxP for KO and suggested that other recombinase recognition sites could also be used.

## 7. Query 4: Are There Tools One Can Use to Check if the Chosen Placement of the rAI in the Gene Will Lead to Correct Splicing or Adequate mRNA Degradation upon Recombinase-Induced Loss of Branchpoints before Committing to Make a Mouse?

There are several free up-to-date splicing prediction tools available that are fairly easy to use for testing one’s design. However, at present, only Spliceator is meant for a wide range of species, including mouse. Programs predicting the effect of rAI or recombinase-treated rAI on a gene for other processes important to the rAI technique, such as NMD, 3′ UTR effect on mRNA degeneration, or branchpoint prediction, are not nearly as advanced or do not exist yet. Note that these tools are never 100% accurate. Spliceator’s accuracy is 89–92% [16], while the rules of the NMDetective program discussed below for predicting NMD account for 68% or 71% of its predictive performance, depending on which version is used [36]. 

*We suggest using an rAI placement in the exon that causes less than a 1% drop in the exon’s Spliceator splicing scores.*

Splice site reliability can be checked by use of an up-to-date mouse- or multi-species-based splice site prediction tool such as Spliceator [16] (http://www.lbgi.fr/spliceator/, last accessed 8 March 2023). Deep learning programs such as Spliceator and SpliceRover have been shown to be highly sensitive to the splice site patterns near the splice site they are investigating, and when they are changed, this can greatly lower the odds that the splice site will still work [37]. The primary predictive difference between how often an alternative splice site will be used and a constitutive splice site used is the reliability of both the donor and acceptor splice site scores [38]. Donor and acceptor splice sites from constitutive exons usually have very high scores, while alternative exons typically have at least one relatively lower splice site score. Any increase or decrease could possibly cause unwanted non-canonical splicing events [39]. The small amount of data we have analyzed (Table 1 and Figure 3) suggests that, upon insertion of the rAI, nearby endogenous splice site scores should decrease at most by about 1% if at all to make sure endogenous splicing is not affected. If scores decrease by more than a few percent, the rAI should be inserted into a different position in the exon or in another exon. 

Spliceator reliability scores were generated from sequences put together using data given in Cassidy et al. [15] and Wu et al. [14] and from our own data (Table 1). In two genes, the Spliceator reliability score of the exon’s SAS went down by more than 1% after insertion of the full-length rAI (Table 1, Exon SAS). For the Phf21a gene, the rAI was placed in a MAG-R sequence 46 nt from the exon’s upstream end, which is in the middle of the region that has a high probability of containing SAS SREs (Figure 3). This resulted in the exon’s SAS score dropping almost 5%. In this case, the rAI did not splice out correctly, instead creating several abnormal splice products. On the other hand, in the Sav1 gene, when the rAI was inserted well away from the critical SRE-containing regions (Figure 3), the Spliceator score dropped 1.6%, and the full-length-rAI-containing homozygous mice, in which KO of this gene is lethal, were fine. This may imply that a drop in score of 1–2% can be tolerated and still lead to correctly functioning rAI, but problems may arise with a drop of 4–5% or more. However, more testing must be performed with these tools to accurately determine how much of a change in score is acceptable after inserting an rAI.

After branchpoint removal, the rAI SAS reliability value changed, almost always downwards (Table 1, rAI SAS). Lower scores are expected, as a splice acceptor must have a branchpoint to function correctly. However, branchpoints are highly degenerate in sequence, so it is difficult to predict which sequences in the exon might be able to act as a branchpoint. Cassidy et al. are the only ones to have sequenced mRNA products from an rAI-containing gene, in this case Scyl1, after branchpoint removal by recombinase. Scyl1′s Spliceator rAI SAS scores show only a 0.1% drop, from 0.999 to 0.998, after rAI branchpoint removal, which may imply that there is an alternative upstream sequence available to act as a branchpoint in lieu of the lost rAI branch point. In support of this, Cassidy et al. did see a splice product that was generated by the strong rAI SAS splicing with a cryptic upstream donor site, albeit at a very low level. In contrast to the SAS scores, the rAI’s SDS values almost always stayed the same after branchpoint removal, changing at most by 0.1% (Table 1, rAI SDS).

The greater the amount and better quality of data a deep learning program has, the more reliable the prediction it can give [40]. Values of splice sites close to an unusual structure such as an extremely long intron may not be predicted as accurately since there are far fewer examples of such regions in the databases used to train these programs. 

*The prediction tools for NMD, 3′ UTR, branchpoint, and mRNA degradation are not as accurate or as easy to use at present as Spliceator. In fact, we suggest that they may not be necessary if our guide is followed, and the planned sequences containing the rAI are checked with Spliceator.*

Although there are some programs for predicting the efficiency of NMD that occurs when a PTC is present, their accuracy is rather low. A surprisingly high percentage of genes with PTCs fail to decay or only partially decay, in turn producing truncated proteins (NMD evasion) despite following standard NMD rules [24]. Perhaps the best tool at present, although more involved to use, is NMDetective [36], which is intended for human and mouse genomes. The simpler, more usable form, NMDetective-B (68% predictive), uses an algorithm consisting of a tree with four nested tests developed from rules obtained by inference from automated data. These tests look for ways for NMD to fail: “(i) if the PTC is in the last exon, (ii) if it is in the last 50 nt of the penultimate exon; (iii) if it is less than 150 nt away from the start codon; and (iv) if it is in a long exon (>407 nt)” [36]. These tests use rules that parallel or are similar to several of the rules we have already discussed here for rAI placement, leading to successful splicing and KO upon recombinase treatment. This suggests that if one follows the rAI placement rules, the use of NMDetector may be superfluous.

For most 3′ UTR prediction programs, including the most current 3′ UTR predictor, F3UTER [41], one can only input a gene identifier [42], so the programs cannot be used to predict the effect of inserting the rAI on 3′ UTRs. One older program, ExUTR [42], is set up to use transcriptome assemblies from any mammal as input and requires installation on a Linus-based operating system.

Current branchpoint predictors appear to be set up primarily for predicting branchpoint detection in human introns [43]. It is not clear how they would function in mouse exons. However, it may not be necessary to use a branchpoint predictor since splice prediction programs are expected to take into account the effects of nearby presumptive branchpoint sequences in their prediction of SAS reliability.

A hybrid convolutional and recurrent deep neural network, Saluki, has been developed recently [44] and predicts mRNA half-life from only its sequence, coding frame, and splice sites. However, its predictive ability at present is only around 59%, and it requires installation from GitHub.

*As splice prediction software and programs predicting the various mRNA degradation processes advance, it may be possible to use them together to increase the success rate of the rAI method. We suggest checking periodically for and then employing these new, updated prediction tools, as this should improve one’s chances of obtaining successful tissue-specific KO when choosing which rAI to use and where to place the rAI.*

## 8. Query 5: What Kind of Donor DNA and Length of Homology Arms Should Be Used?

*The usual CRISPR-Cas9 rules apply. Because the rAIs are short, ssDNA and short homology arms can work well if the CRISPR-Cas9 cut site is within 10 bp.*

In our attempt to create a Phf21a tissue-specific KO mouse using DECAI, we were able to obtain a good insertion rate (5 out of 14 blastocysts and 3 out of 18 mice) with ssDNA and homology arms of 37 nt on the upstream side of the DECAI intron and 100 nt on the downstream side. The cut site was 5 bp from the insertion site. Both Cassidy et al. [15] and Wu et al. [14] also had high rates of perfect insertion using single-stranded DNA donors. Cassidy et al. used 58 nt and 60 nt length arms and Wu et al. 55 nt and 56 nt arms upstream and downstream, respectively. Results are expected to vary for different insertion sites and genes depending on how well a guide directs a cut, how far the cut site is from the insertion site, and other factors known to effect CRISPR-Cas9.

## 9. Discussion 

In this paper, we have provided evidence from the literature that suggests one can obtain successful tissue-specific KO with an rAI by adhering to the following main points:

1. Use an rAI such as SCON, whose length in bps is not divisible by 3;
2. Insert the rAI into a constitutive exon that is preferably followed by another constitutive exon. The closer such an exon is to the transcription start site in the mRNA, the better, as long as it is still at least ~200 bp away from the start codon;
3. Place the rAI within the exon as close to the downstream (3′) end as is feasible while avoiding the first and last ~100 bp of the exon and staying within the first 400 bp of the exon 3′ end;
4. Insert the rAI such that if the rAI donor site splices with the downstream exon acceptor site, a frameshift will occur;
5. Place the rAI into a sequence such as MAG-R, which produces high rAI splice donor and acceptor site scores from an up-to-date splicing prediction program such as Spliceator;
6. Additionally, the rAI placement should affect scores of potential and actual endogenous splice sites at most only slightly (<1% for Spliceator), if at all.

However, the instructions we have presented are subject to changes and improvements as more information becomes available. For this reason, this is an interim guide that still has several caveats. 

An inability to follow the rules and suggestions in this guideline may indicate that certain genes might not be appropriate for the rAI approach. On the other hand, some of the instructions, for example, in Query 2, namely keeping the rAI insertion away from the exon ends, are set up in a more general way because splicing is too complicated to apply every single known factor individually when deciding whether the rAI approach will work. It might be possible to “bend” these rules by testing with Spliceator to check if placement in an inappropriate position is likely to affect splicing. However, no data are available yet to determine if this can be done.

It is also important to keep in mind that even if one is able to follow all of the guidelines, problems may arise. Spliceator is not 100% guaranteed to be reliable. Despite placing the rAI in a position that upon recombinase treatment should produce a frameshift within all expected splice products, the planned outcome may not occur. Various other factors such as RNA secondary structure, splicing regulatory elements, post-transcriptional modification of nucleotides, trans-acting factors, and histone modifications regulating transcription can all affect the outcome in ways that at present are difficult to predict [34], and unexpected splice products may still be created. Cell type and stress level, tissue type, stage of development, etc., can also affect splicing differently [45,46,47,48].

Furthermore, the extent of NMD caused by a PTC in a gene can vary widely between different species, different tissues in an organism, and different cells in the same tissue [49]. Certain cell processes controlling gene expression can be refractory to mRNA-based degradation mechanisms. For example, some genes are resistant to mRNA reduction by NMD. One common reason for resistance in these genes is a feedback mechanism, namely generating more mRNA to keep the level constant (dosage compensated genes [18]), as occurs for most X-linked gene products. These kinds of issues must be determined on a case-by-case basis. 

Since the final amount of each differently spliced mRNA product is a combination of the levels of its assembly and degradation, it is impossible at this time to predict the extent to which various created mRNA products might exist. Thus, it is prudent to check for the level of both protein and mRNA production from an rAI-containing gene before and after crossing with the appropriate recombinase-expressing mouse. 

In comparing the rAI method with the standard method of floxing by placing loxPs into the two introns flanking an essential exon, inappropriate positioning of these loxPs in the intron can also affect splicing. There are also other very important, newly discovered functions of the intron, such as the enhancement of protein expression [50,51], that can be disrupted by inserting loxPs into the wrong position. At present, there does not seem to be a similar in-depth guide as proposed here that explains where is best to place the loxPs into an intron to prevent functional alterations.

As more is discovered about the limitations to proper splicing, and as better splice prediction and prediction tools for RNA degradation are developed, the success rate when using this alternative new technique for tissue-specific KO should continue to rise.

## Figures and Tables

**Table 1:** Spliceator splice site reliability scores for the exon and rAI splice acceptor site (SAS) and splice donor site (SDS) of the exons shown in Figure 3. Scores were generated from sequences before rAI insertion (WT), after insertion (rAI), and after insertion and treatment with recombinase (rAI+R). Exon SAS scores that changed more than 1% after rAI insertion into the gene but before recombinase treatment are shown in red.

	exon SAS			rAI SDS		rAI SAS		exon SDS		
	WT	rAI	rAI+R	rAI	rAI+R	rAI	rAI+R	WT	rAI	rAI+R
Phf21a exon 4	0.981	0.935	<0.85	0.999	0.998	0.995	0.996	0.999	0.999	0.999
Abi3 exon 3	0.881	0.883	<0.85	1	0.999	0.981	0.958	0.997	0.996	0.998
Ace2 exon 2	0.996	0.996	0.994	0.998	0.999	0.992	0.988	0.996	0.993	0.996
Lpar1 exon 2	0.995	0.987	0.99	0.999	0.999	0.988	0.972	0.998	0.998	0.998
Usp42 exon 2	0.994	0.991	0.989	0.999	0.999	0.968	0.927	0.998	0.995	0.996
Zmpste24 exon 7	0.993	0.983	0.978	0.999	0.999	0.985	0.978	0.997	0.996	0.993
Mlh1 exon 12	0.993	0.995	0.994	1	1	0.999	0.998	1	1	1
Sav1 exon 2	0.977	0.961	0.97	0.999	0.999	0.989	0.985	0.998	0.998	0.998
Ctnnb1 exon 4	0.983	0.982	0.98	0.999	0.999	0.994	0.98	0.977	0.98	0.987
Cdh12 exon 2	0.998	0.998	0.998	0.999	0.999	0.996	0.997	0.987	0.988	0.988
Rnf34 exon 3	0.953	0.973	0.971	1	1	0.991	0.99	0.998	0.998	0.999
Scyl1 exon 3	0.991	0.995	0.997	0.996	0.996	0.999	0.998	0.986	0.992	0.992
Lpar2 exon 1	exon 1, no splice site			1	1	0.999	0.997	0.983	0.983	0.983
Tert exon 2	0.995	0.995	0.995	0.909	0.909	0.992	0.983	0.971	0.971	0.971
Sox2 single exon	single exon, no splice sites			1	1	0.999	0.999	single exon, no splice sites		

## Data Availability

Not applicable.

## Abbreviations

bp	Base pair
Cas9	CRISPR-associated protein 9
CRISPR	Clustered regularly interspaced short palindromic repeats
DECAI	Degradation based on Cre-regulated artificial intron
eGFP	Enhanced green fluorescent protein
EJC	Exon junction complex
hnRNP	Heterogeneous ribonucleoprotein
KO	Knockout
Long 3′ UTR	Long 3′ untranslated region
IssDNA	Long single-strand DNA
NMD	Nonsense-mediated decay
nt	Nucleotide
PTC	Premature termination codon
rAI	Recombinase-regulated artificial intron
SAS	Splice acceptor site
SCON	Short conditional intron
SDS	Splice donor site
snRNA	Small nuclear RNA
SR	Serine- and arginine-rich protein
SRE	Splicing regulatory element

## References

[1] Gurumurthy C.B., O’Brien A.R., Quadros R.M., Adams J., Alcaide P., Ayabe S., Ballard J., Batra S.K., Beauchamp M.-C., Becker K.A. (2019). Reproducibility of CRISPR-Cas9 methods for generation of conditional mouse alleles: A multi-center evaluation. Genome Biol..

[2] Horii T., Morita S., Kimura M., Terawaki N., Shibutani M., Hatada I. (2017). Efficient generation of conditional knockout mice via sequential introduction of lox sites. Sci. Rep..

[3] Gu B., Posfai E., Rossant J. (2018). Efficient generation of targeted large insertions by microinjection into two-cell-stage mouse embryos. Nat. Biotechnol..

[4] Shang R., Zhang H., Bi P. (2021). Generation of mouse conditional knockout alleles in one step using the i-GONAD method. Genome Res..

[5] Bernas G., Ouellet M., Barrios A., Jamann H., Larochelle C., Lévy É., Schmouth J.-F. (2022). Introduction of loxP sites by electroporation in the mouse genome; a simple approach for conditional allele generation in complex targeting loci. BMC Biotechnol..

[6] Lanza D.G., Gaspero A., Lorenzo I., Liao L., Zheng P., Wang Y., Deng Y., Cheng C., Zhang C., Rasband M.N. (2017). Employing single-stranded DNA donors for the high-throughput production of conditional knockout alleles in mice. bioRxiv.

[7] Lanza D.G., Gaspero A., Lorenzo I., Liao L., Zheng P., Wang Y., Deng Y., Cheng C., Zhang C., Seavitt J.R. (2018). Comparative analysis of single-stranded DNA donors to generate conditional null mouse alleles. BMC Biol..

[8] Quadros R.M., Miura H., Harms D.W., Akatsuka H., Sato T., Aida T., Redder R., Richardson G.P., Inagaki Y., Sakai D. (2017). Easi-CRISPR: A robust method for one-step generation of mice carrying conditional and insertion alleles using long ssDNA donors and CRISPR ribonucleoproteins. Genome Biol..

[9] Codner G.F., Mianné J., Caulder A., Loeffler J., Fell R., King R., Allan A.J., Mackenzie M., Pike F.J., McCabe C.V. (2018). Application of long single-stranded DNA donors in genome editing: Generation and validation of mouse mutants. BMC Biol..

[10] Miyasaka Y., Uno Y., Yoshimi K., Kunihiro Y., Yoshimura T., Tanaka T., Ishikubo H., Hiraoka Y., Takemoto N., Tanaka T. (2018). CLICK: One-step generation of conditional knockout mice. BMC Genom..

[11] Chen S., Sun S., Moonen D., Lee C., Lee A.Y.-F., Schaffer D.V., He L. (2019). CRISPR-READI: Efficient Generation of Knockin Mice by CRISPR RNP Electroporation and AAV Donor Infection. Cell Rep..

[12] Tanimoto Y., Mikami N., Ishida M., Iki N., Kato K., Sugiyama F., Takahashi S., Mizuno S. (2022). Zygote Microinjection for Creating Gene Cassette Knock-in and Flox Alleles in Mice. JoVE (J. Vis. Exp.).

[13] Guzzardo P.M., Rashkova C., dos Santos R.L., Tehrani R., Collin P., Bürckstümmer T. (2017). A small cassette enables conditional gene inactivation by CRISPR/Cas9. Sci. Rep..

[14] Wu S.-H.S., Lee H., Szép-Bakonyi R., Colozza G., Boese A., Gert K.R., Hallay N., Lee J.-H., Kim J., Zhu Y. (2022). SCON—A Short Conditional intrON for conditional knockout with one-step zygote injection. Exp. Mol. Med..

[15] Cassidy A.M., Thomas D.B., Kuliyev E., Chen H., Pelletier S. (2022). One-step generation of a conditional allele in mice using a short artificial intron. Heliyon.

[16] Scalzitti N., Kress A., Orhand R., Weber T., Moulinier L., Jeannin-Girardon A., Collet P., Poch O., Thompson J.D. (2021). Spliceator: Multi-species splice site prediction using convolutional neural networks. BMC Bioinform..

[17] Rivas M.A., Pirinen M., Conrad D.F., Lek M., Tsang E.K., Karczewski K.J., Maller J.B., Kukurba K.R., DeLuca D.S., Fromer M. (2015). Effect of predicted protein-truncating genetic variants on the human transcriptome. Science.

[18] Lindeboom R.G.H., Supek F., Lehner B. (2016). The rules and impact of nonsense-mediated mRNA decay in human cancers. Nat. Genet..

[19] Embree C.M., Abu-Alhasan R., Singh G. (2022). Features and factors that dictate if terminating ribosomes cause or counteract nonsense-mediated mRNA decay. J. Biol. Chem..

[20] Spasic M., Friedel C.C., Schott J., Kreth J., Leppek K., Hofmann S., Ozgur S., Stoecklin G. (2012). Genome-Wide Assessment of AU-Rich Elements by the AREScore Algorithm. PLoS Genet..

[21] Otsuka H., Fukao A., Funakami Y., Duncan K.E., Fujiwara T. (2019). Emerging Evidence of Translational Control by AU-Rich Element-Binding Proteins. Front. Genet..

[22] Hurt J.A., Robertson A.D., Burge C.B. (2013). Global analyses of UPF1 binding and function reveal expanded scope of nonsense-mediated mRNA decay. Genome Res..

[23] Inácio Â., Silva A.L., Pinto J., Ji X., Morgado A., Almeida F., Faustino P., Lavinha J., Liebhaber S.A., Romão L. (2004). Nonsense Mutations in Close Proximity to the Initiation Codon Fail to Trigger Full Nonsense-mediated mRNA Decay. J. Biol. Chem..

[24] Supek F., Lehner B., Lindeboom R.G.H. (2021). To NMD or Not To NMD: Nonsense-Mediated mRNA Decay in Cancer and Other Genetic Diseases. Trends Genet..

[25] Karousis E.D., Mühlemann O. (2022). The broader sense of nonsense. Trends Biochem. Sci..

[26] Busch A., Hertel K.J. (2015). Splicing predictions reliably classify different types of alternative splicing. RNA.

[27] Herzel L., Ottoz D.S.M., Alpert T., Neugebauer K.M. (2017). Splicing and transcription touch base: Co-transcriptional spliceosome assembly and function. Nat. Rev. Mol. Cell Biol..

[28] Popp M.W., Maquat L.E. (2016). Leveraging Rules of Nonsense-Mediated mRNA Decay for Genome Engineering and Personalized Medicine. Cell.

[29] Nogueira G., Fernandes R., García-Moreno J.F., Romão L. (2021). Nonsense-mediated RNA decay and its bipolar function in cancer. Mol. Cancer.

[30] Mercer T.R., Clark M.B., Andersen S.B., Brunck M.E., Haerty W., Crawford J., Taft R.J., Nielsen L.K., Dinger M.E., Mattick J.S. (2015). Genome-wide discovery of human splicing branchpoints. Genome Res..

[31] Zhang P., Philippot Q., Ren W., Lei W.-T., Li J., Stenson P.D., Palacín P.S., Colobran R., Boisson B., Zhang S.-Y. (2022). Genome-wide detection of human variants that disrupt intronic branchpoints. Proc. Natl. Acad. Sci. USA.

[32] Taggart A.J., Lin C.-L., Shrestha B., Heintzelman C., Kim S., Fairbrother W.G. (2017). Large-scale analysis of branchpoint usage across species and cell lines. Genome Res..

[33] Burset M., Seledtsov I.A., Solovyev V.V. (2000). Analysis of canonical and non-canonical splice sites in mammalian genomes. Nucleic Acids Res..

[34] Borao S., Ayté J., Hümmer S. (2021). Evolution of the Early Spliceosomal Complex—From Constitutive to Regulated Splicing. Int. J. Mol. Sci..

[35] Hartmann L., Theiss S., Niederacher D., Schaal H. (2008). Diagnostics of pathogenic splicing mutations: Does bioinformatics cover all bases?. FBL.

[36] Lindeboom R.G.H., Vermeulen M., Lehner B., Supek F. (2019). The impact of nonsense-mediated mRNA decay on genetic disease, gene editing and cancer immunotherapy. Nat. Genet..

[37] Zuallaert J., Godin F., Kim M., Soete A., Saeys Y., De Neve W. (2018). SpliceRover: Interpretable convolutional neural networks for improved splice site prediction. Bioinformatics.

[38] Müller L., Ptok J., Nisar A., Antemann J., Grothmann R., Hillebrand F., Brillen A.-L., Ritchie A., Theiss S., Schaal H. (2022). Modeling splicing outcome by combining 5′ss strength and splicing regulatory elements. Nucleic Acids Res..

[39] Sibley C.R., Blazquez L., Ule J. (2016). Lessons from non-canonical splicing. Nat. Rev. Genet..

[40] Cortes C., Jackel L.D., Chiang W.-P. (1994). Limits on Learning Machine Accuracy Imposed by Data Quality. Advances in Neural Information Processing Systems.

[41] Sethi S., Zhang D., Guelfi S., Chen Z., Garcia-Ruiz S., Olagbaju E.O., Ryten M., Saini H., Botia J.A. (2022). Leveraging omic features with F3UTER enables identification of unannotated 3′ UTRs for synaptic genes. Nat. Commun..

[42] Huang Z., Teeling E.C. (2017). ExUTR: A novel pipeline for large-scale prediction of 3′-UTR sequences from NGS data. BMC Genom..

[43] Leman R., Tubeuf H., Raad S., Tournier I., Derambure C., Lanos R., Gaildrat P., Castelain G., Hauchard J., Killian A. (2020). Assessment of branch point prediction tools to predict physiological branch points and their alteration by variants. BMC Genom..

[44] Agarwal V., Kelley D.R. (2022). The genetic and biochemical determinants of mRNA degradation rates in mammals. Genome Biol..

[45] Olivieri J.E., Dehghannasiri R., Wang P.L., Jang S., de Morree A., Tan S.Y., Ming J., Ruohao Wu A., Quake S.R., Tabula Sapiens Consortium (2021). RNA splicing programs define tissue compartments and cell types at single-cell resolution. eLife.

[46] Shiina T., Shimizu Y. (2020). Temperature-Dependent Alternative Splicing of Precursor mRNAs and Its Biological Significance: A Review Focused on Post-Transcriptional Regulation of a Cold Shock Protein Gene in Hibernating Mammals. Int. J. Mol. Sci..

[47] Mazin P.V., Khaitovich P., Cardoso-Moreira M., Kaessmann H. (2021). Alternative splicing during mammalian organ development. Nat. Genet..

[48] Ullah F., Jabeen S., Salton M., Reddy A.S.N., Ben-Hur A. (2023). Evidence for the role of transcription factors in the co-transcriptional regulation of intron retention. Genome Biol..

[49] Sato H., Singer R.H. (2021). Cellular variability of nonsense-mediated mRNA decay. Nat. Commun..

[50] Rose A.B. (2019). Introns as Gene Regulators: A Brick on the Accelerator. Front. Genet..

[51] Chorev M., Joseph Bekker A., Goldberger J., Carmel L. (2017). Identification of introns harboring functional sequence elements through positional conservation. Sci. Rep..

